# Common Kinetic Mechanism of Abasic Site Recognition by Structurally Different Apurinic/Apyrimidinic Endonucleases

**DOI:** 10.3390/ijms22168874

**Published:** 2021-08-18

**Authors:** Alexandra A. Kuznetsova, Svetlana I. Senchurova, Alexander A. Ishchenko, Murat Saparbaev, Olga S. Fedorova, Nikita A. Kuznetsov

**Affiliations:** 1 Institute of Chemical Biology and Fundamental Medicine, Siberian Branch of Russian Academy of Sciences, 630090 Novosibirsk, Russia; sandra-k@niboch.nsc.ru (A.A.K.); s.senchurova@g.nsu.ru (S.I.S.); 2 Department of Natural Sciences, Novosibirsk State University, 630090 Novosibirsk, Russia; 3 Group Mechanisms of DNA Repair and Carcinogenesis, Equipe Labellisée LIGUE 2016, CNRS UMR9019, Gustave Roussy Cancer Campus, Université Paris-Saclay, F-94805 Villejuif, France; Alexander.ISHCHENKO@gustaveroussy.fr (A.A.I.); murat.saparbaev@gustaveroussy.fr (M.S.)

**Keywords:** DNA repair, apurinic/apyrimidinic endonuclease, damaged DNA, abasic site, conformational dynamics, stopped-flow enzyme kinetics

## Abstract

Apurinic/apyrimidinic (AP) endonucleases Nfo (*Escherichia coli*) and APE1 (human) represent two conserved structural families of enzymes that cleave AP-site–containing DNA in base excision repair. Nfo and APE1 have completely different structures of the DNA-binding site, catalytically active amino acid residues and catalytic metal ions. Nonetheless, both enzymes induce DNA bending, AP-site backbone eversion into the active-site pocket and extrusion of the nucleotide located opposite the damage. All these stages may depend on local stability of the DNA duplex near the lesion. Here, we analysed effects of natural nucleotides located opposite a lesion on catalytic-complex formation stages and DNA cleavage efficacy. Several model DNA substrates that contain an AP-site analogue [F-site, i.e., (2*R*,3*S*)-2-(hydroxymethyl)-3-hydroxytetrahydrofuran] opposite G, A, T or C were used to monitor real-time conformational changes of the tested enzymes during interaction with DNA using changes in the enzymes’ intrinsic fluorescence intensity mainly caused by Trp fluorescence. The extrusion of the nucleotide located opposite F-site was recorded via fluorescence intensity changes of two base analogues. The catalytic rate constant slightly depended on the opposite-nucleotide nature. Thus, structurally different AP endonucleases Nfo and APE1 utilise a common strategy of damage recognition controlled by enzyme conformational transitions after initial DNA binding.

## 1. Introduction

Recognition and removal of non-bulky damaged nitrogenous bases from DNA proceed via the base excision repair (BER) pathway initiated by DNA glycosylases [1,2,3]. By cleaving an apurinic/apyrimidinic (AP)-site and by removing a blocking 3′-end group such as 3′-phospho-α,β-unsaturated aldehyde or 3′-terminal phosphate, AP endonucleases process the intermediate DNA products generated by DNA glycosylases [4]. AP endonucleases also can recognise some damaged nucleotides containing a modified base, for example, 5,6-dihydrouracil, the α-anomer of adenosine or several others [5,6,7,8,9,10,11] and initiate DNA glycosylase–independent nucleotide incision repair (NIR) [12,13,14]. In addition, these enzymes possess endoribonuclease [15,16,17], 3′-phosphodiesterase, 3′-phosphatase [18] and 3′→5′ exonuclease activities [19,20,21].

**Figure 1:**
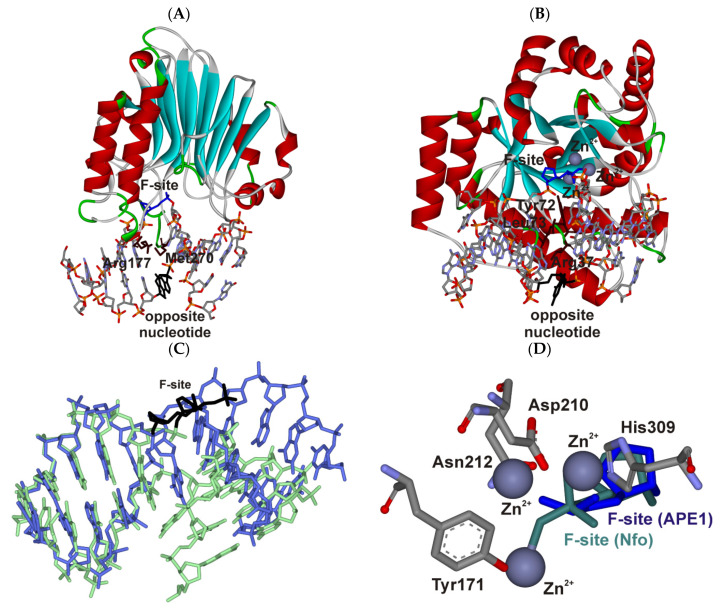
Conformational rearrangements of DNA bound by APE1 (Protein Data Bank ID: 1DE8) (**A**) or Nfo (Protein Data Bank ID: 1QUM) (**B**). The damaged nucleotide is blue, the complementary nucleotide is black and ‘void-filling’ amino acids are highlighted in brown. Structural superposition of DNA bound by APE1 (blue, 1DE8) and Nfo (green, 1QUM) aligned by part of DNA backbone (**C**). Structural superposition of active sites of APE1 (1DE8) and Nfo (1QUM) aligned by F-site coordinates (**D**). Residues Tyr171, Asp210, Asn212 and His309 of human APE1 are functionally equivalent to the three Zn^2+^ ions of Nfo.

AP endonucleases are categorised into two structural families based on their similarity to prokaryotic exonuclease III (Exo III or Xth, Figure 1A) or endonuclease IV (Endo IV or Nfo, Figure 1B) [22,23,24,25]. Members of the Xth family have a bilayer β-sheet flanked by α-helices and are Mg^2+^-dependent enzymes. Members of the Nfo family have a structural core in the form of a β-barrel surrounded by α-helices and contain three Zn^2+^ ions in the active site. According to structural data [24,26], in the Nfo complex with DNA containing an AP-site analogue [F-site, i.e., (2*R*,3*S*)-2-(hydroxymethyl)-3-hydroxytetrahydrofuran], the ribose-phosphate backbone of the duplex is bent by ~90° (Figure 1C, green), and the damaged nucleotide is inserted into the active site of the enzyme. In addition, the nucleotide opposite the lesion is flipped out of the double helix. On the contrary, in an APE1–DNA complex [27], the angle of ribose-phosphate backbone bending is only ~35° (Figure 1C, blue). In this case, the damaged nucleotide is also inserted into the active site, but the nucleotide located opposite the damage retains its stacking with neighbouring bases in the DNA duplex. Despite differences in the structure and metal ion requirements of APE1 and Nfo (Figure 1D), a comparison of their active-site structures surprisingly reveals functional equivalence between Nfo Zn^2+^ ions and APE1 catalytic amino acid residues Tyr171, Asp210, Asn212 and His309 [25,28]. Moreover, both enzymes insert some residues (Arg177 and Met207 [APE1] and Arg37, Tyr72 and Leu73 [Nfo], Figure 1A,B) into the void formed in the DNA duplex after damage eversion. These ‘void-filling’ amino acids can act both as a specific ‘wedge’ sensing the lesion and as non-specific anchor residues that stabilise the extra-helical position of the damaged nucleotide.

It can be assumed that non-specific contacts between amino acid residues of the DNA-binding site and DNA backbone serve to sculpt DNA structure for bending the double helix, local DNA melting and damaged-nucleotide eversion. The ability of DNA to be bent by the enzyme is related to the flexibility of the double helix. A damaged nucleotide can increase double-helix flexibility or even give rise to stable curvature of DNA, depending on the nature of the nucleobase located opposite the damage. The enzyme-induced distorted state of the DNA helix promotes initial eversion of the damaged nucleotide, and this process may be facilitated by ‘wedging’ interactions with some of active-site amino acid residues. Of note, such non-specific interactions are mostly independent of the nature of the damaged nucleotide and therefore cannot significantly influence the damage recognition process. In contrast, the nature of the base opposite to the damaged nucleotide may affect the efficacy of the damage eversion owing to an influence on the stability of Watson–Crick hydrogen bonding and on the flexibility of the duplex at the damage site. Therefore, it is important to analyse the effects of natural nucleotides situated opposite the damage on the separate stages of catalytic-complex formation and on the efficacy of DNA cleavage by AP endonucleases APE1 and Nfo. For this purpose, we studied real-time conformational changes of the enzymes during their interaction with damaged DNA containing an abasic site (F-site); these changes were monitored by means of changes in Trp fluorescence intensity. The comparative analysis of the cleavage of the model DNA substrates by AP endonucleases from Nfo and Xth structural families allowed us to elucidate the mechanism of catalytic-complex formation and to reveal the key steps that affected abasic-site recognition.

## 2. Results and Discussion

### 2.1. The Rationale

The mechanism of recognition of abasic site by AP endonucleases includes DNA bending, damaged-nucleotide eversion from the double helix and insertion into the active-site pocket of the enzyme [24,26,27]. It could be hypothesise that the efficiency of the DNA bending and abasic site eversion stages can be determined by a few factors: (i) the formation of network contacts with the DNA backbone upstream and downstream of the lesion, (ii) direct ‘pushing’ or ‘pulling’ interactions with the damaged nucleotide, (iii) possible steric hindrance between the 2′-deoxyribose moiety and active-site amino acid residues in the course of the eversion and (iv) the influence of the complementary-nucleotide nature on the ability of the DNA to bend at the damage site.

Structural data permits the assumption that non-specific contacts between DNA-binding site amino acids and the DNA backbone sculpt DNA structure thereby possibly bending the double helix. This enzyme-induced DNA helix distortion promotes the initial flipping of the damaged nucleotide. It is possible that these interactions are mostly independent from the nature of the damaged nucleotide and consequently cannot significantly influence the efficiency of catalytic-complex formation. On the other hand, the opposite base may affect the efficacy of the damaged-nucleotide eversion because of its influence on the energetics of complementary interactions with the lesion and on local flexibility of the duplex near the damaged pair. Moreover, a conformational rearrangement of DNA during the search for and/or specific recognition of the damaged nucleotide can be facilitated by the emergence of direct contacts with the nucleotide located opposite the damage or through inner interactions of the opposite base with neighbouring bases in the double helix. Therefore, the nature of the opposite nucleotide can have an impact on the processes of DNA bending, damage eversion and eventually catalytic-complex formation.

### 2.2. Conformational Changes of AP Endonucleases in the Course of F/N-Substrate Cleavage

**Figure 2:**
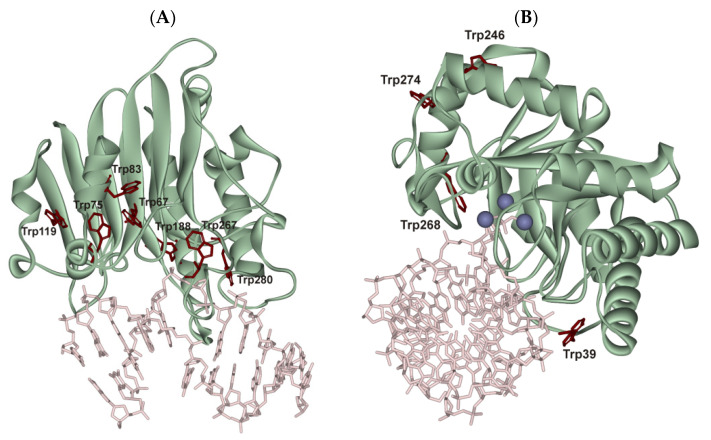
Distribution of Trp residues in spatial structures of human APE1 (**A**) and Nfo from *Escherichia coli* (**B**).

Due to the recognition of specific sites in real time accompanied by a conformational adjustment of an enzyme molecule and DNA for optimisation of specific contacts, we performed a pre–steady-state kinetic analysis of conformational changes of the AP endonucleases and model DNA substrates during the enzyme–substrate interaction. Conformational changes in the enzyme molecules were monitored via changes in Trp fluorescence intensity. It has been shown [29,30,31] that despite the rigid protein core of APE1, changes in Trp fluorescence intensity allow us to identify the steps of DNA binding, cleavage and product release. It was likely that one of APE1′s seven Trp residues (Figure 2A, Trp280), which is located in the DNA-binding pocket of the enzyme and engages in a hydrogen bond with the 3′-phosphate group of the AP-site, is responsible for the observed changes in fluorescence. Nfo contains four Trp residues distributed in the structure (Figure 2B). It should be noted that one of them, Trp39, binds to the phosphate groups of the nucleotide placed opposite the AP-site, which is everted from the double helix in the catalytic complex. Therefore, the changes in protein fluorescence probably characterise the conformational changes in the Trp39 environment and could be monitored for identifying the extruded nucleotide.

**Scheme 1:**
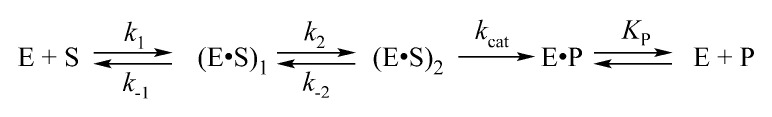
The kinetic mechanism underlying the processing of F/N-substrates by the AP endonucleases as revealed by Trp fluorescence intensity detection. E is AP endonuclease; S denotes an F/N-substrate; P means the product of the substrate incision reaction; (E•S)1 is an initial pre-catalytic enzyme–substrate complex; (E•S)2 is a catalytically active enzyme–substrate complex; E•P is a complex of the enzyme with the product; *k*_1_, *k*_-1_, *k*_2_ and *k*_-2_ are rate constants of equilibrium steps; and *k*_cat_ stands for the rate constant of 5′ phosphodiester bond catalytic cleavage.

It has been reported [29] that kinetic traces of APE1′s reaction with DNA containing an F-site opposite G (F/G-substrate) comprise the characteristic phases of a decrease and an increase in Trp fluorescence intensity, which can be assigned to DNA-binding steps and catalysis steps, respectively. It was shown that during the recognition of the abasic site in an F/G-substrate, at least two steps of conformational transition occur in the enzyme toward catalytic-complex formation (Scheme 1). In this complex, APE1 excises the phosphodiester bond on the 5′ side of the abasic site. The last step of the kinetic mechanism is the product release. As reported in ref. [32], the formation of the primary enzyme–substrate complex in Scheme 1 is characterised by a positive value of standard entropy change, which was attributed to the desolvation of polar groups in the protein–DNA contact area and to a release of water molecules from DNA grooves, corresponding to transition from highly ordered pseudo-crystalline state to disordered state. This step may include a non-specific interaction between the phosphate groups of the DNA duplex on the 5′ and 3′ sides of the F-site and some amino acid residues of the DNA-binding site, e.g., Arg73, Ala74, Lys78, Trp280, Asn222, Asn226 and Asn229. Moreover, it was assumed that at this moment, Arg177 and Met270 insertion takes place, which is accompanied by the displacement of water molecules in DNA grooves. At the second step of the interaction of APE1 with an F/G-substrate, a specific rearrangement of the primary complex occurs, which includes the F-site eversion into the enzyme active site.

**Figure 3:**
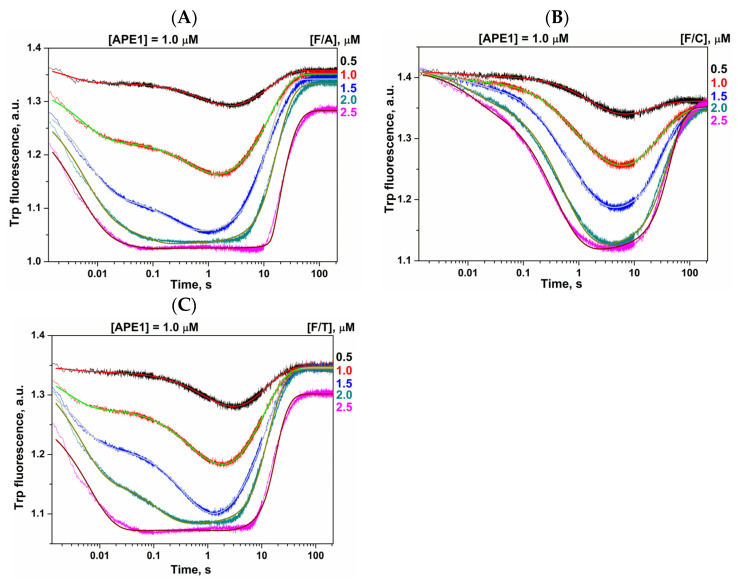
Experimental and theoretical kinetic curves characterising the changes in Trp fluorescence intensity during the interaction of APE1 with an F/N-substrate containing adenosine (**A**), cytidine (**B**) or thymidine (**C**) opposite the F-site. [APE1] = 1.0 μM.

To reveal the impact of the opposite nucleotide on the F-site recognition and catalysis, we used the set of F/N pairs where N = A, C or T (Figure 3). The obtained kinetic traces revealed that the opposite nucleotide significantly affects the kinetic profile of the Trp fluorescence changes during DNA binding. The initial biphasic decrease in Trp fluorescence intensity, revealing the emergence of two pre-excision enzyme–substrate complexes, is distinct for the F/C-substrate. Scheme 1 was employed to fit the observed changes in Trp fluorescence intensity and enabled us to calculate the rate constants of individual reaction steps (Table 1).

Global fitting showed that binding constant *K*_1_ of the first DNA-binding step decreases in the order F/G > F/A ≈ F/T > F/C mostly owing to the destabilisation of the (E•S)_1_ complex by a significant increase in *k*_−1_. In contrast, for the F/C pair, *k*_1_ was also 16-fold lower as compared with the F/G pair. It is noteworthy that the difference in parameters of the second step is not well pronounced and binding constant *K*_2_ of this step is slightly higher for pyrimidine bases than for purine bases. Moreover, this effect is mostly related to reduced *k*_−2_, suggesting that the kinked state of the DNA duplex with the everted F-site is stabler in the case of both pyrimidine bases in comparison with the F/G pair. Nonetheless, a comparison of the total binding constants pointed to the same order of substrate-binding strength: F/G > F/A ≈ F/T > F/C. The rate constant of the catalytic step varied in a narrow range from 0.1 to 0.3 s^−1^, suggesting that the catalytic reaction is independent of the complementary-nucleotide nature. Taken together, this data imply that the complementary nucleotide affects both steps of DNA binding but not the catalytic reaction.

**Figure 4:**
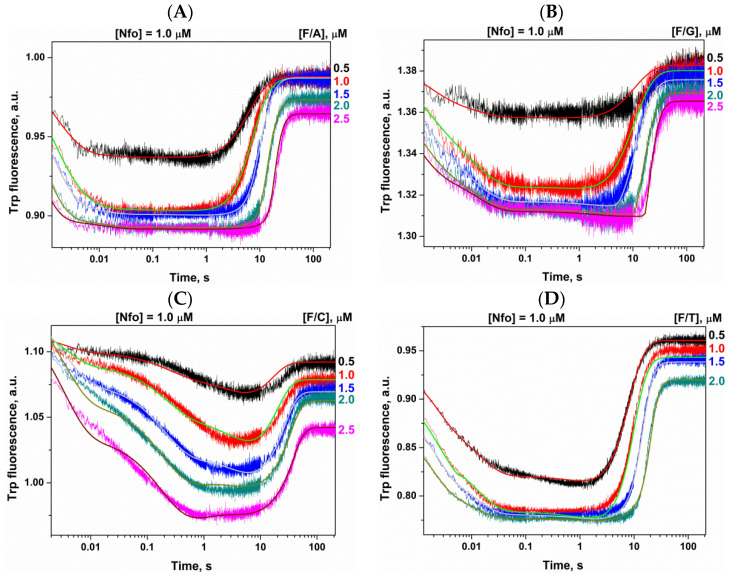
Experimental and theoretical kinetic curves characterising the changes in Trp fluorescence intensity during the interaction of Nfo with an F/N-substrate containing guanosine (**A**), adenosine (**B**), cytidine (**C**) or thymidine (**D**) opposite the F-site. [Nfo] = 1.0 μM.

In the course of the Nfo interaction with a duplex containing the F-site, a sequential decrease and increase in Trp fluorescence intensity was observed (Figure 4). Based on the results on APE1, it was likely that the initial decrease in Trp fluorescence characterises the formation of the enzyme–substrate complex. The catalytic stage of the reaction and the dissociation of the enzyme–product complex is accompanied by a subsequent increase in fluorescence intensity of Trp at time points after 10 s. The profile of the decrease phase of fluorescence intensity significantly depends on the nucleotide situated opposite to the F-site. Thus, for the F/G-substrate and F/A-substrate, which contain a purine nucleotide opposite the damage, the decrease phase proceeds quickly, within 10–30 ms (Figure 4A,B). In the case of DNA substrates containing a pyrimidine nucleotide opposite the F-site, after an initial fast decrease of fluorescence intensity, the second slow decrease phase transpired, which was visualised well for the F/C-substrate (Figure 4C). To note, the amplitude of the decreasing phase is 2–3-fold smaller for the purine orphan base, implying a decrease in the signal-to-noise ratio.

**Scheme 2:**
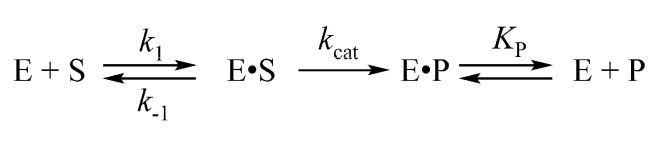
The kinetic mechanism behind the processing of the F/G-substrate or F/A-substrate by Nfo. E is an AP endonuclease, S denotes an F/N-substrate, P represents the product of the substrate incision reaction, E•S is a catalytically active enzyme–substrate complex, E•P means a complex of the enzyme with the product, *k*_1_ and *k*_−1_ are rate constants of equilibrium steps and *k*_cat_ stands for the rate constant of catalytic cleavage of the 5′ phosphodiester bond.

Examination of the kinetic curves suggested that the interaction of Nfo with the F/G-substrate or F/A-substrate is described by a kinetic mechanism involving only one binding step (Scheme 2). On the contrary, for the F/C-substrate and F/T-substrate, the observed kinetic curves were best fitted by Scheme 1, in which the second DNA-binding step corresponds to the slow decrease phase in the kinetic traces. It could be assumed that the smaller signal-to-noise ratio did not permit the resolution of parameters of the second step for purine bases. The rate constants for forward and reverse reactions are given in Table 2. These data revealed that the efficiency of the DNA-binding step, characterised by equilibrium constant *K*_1_, decreases in the order F/G > F/A > F/T > F/C owing to significant elevation of reverse rate constant *k*_−1_ from 1.9 s^−1^ for the F/G pair to 270 s^−1^ for F/C. A comparison of constants of the second phase monitored in the case of pyrimidine bases uncovered a similar equilibrium constant *K*_2_ for both the F/T-substrate and F/C-substrate. Analysis of rate constants suggests that the second step is very fast (*k*_2_ = 101 s^−1^) for the F/T-substrate. When the F/C-substrate was used, the rate constant of the second step was lower by a factor of ~10 (*k*_2_ = 9.1 s^−1^), thereby leading to an extension and shift of the Trp fluorescence intensity decreasing phase up to 1 s. It is likely that for purine bases, the molecular events during this step proceed even faster and therefore cannot be detected as separate steps in the kinetic mechanism. The total binding constants decrease in the order F/G > F/A > F/T > F/C too. The catalytic rate constant also varied in a narrow range from 0.10 to 0.27 s^−1^, just as the constant for APE1, thereby implying independence of the hydrolysis reaction on the opposite-nucleotide nature.

### 2.3. Direct PAGE Analysis of F/N-Substrate Cleavage

**Scheme 3:**
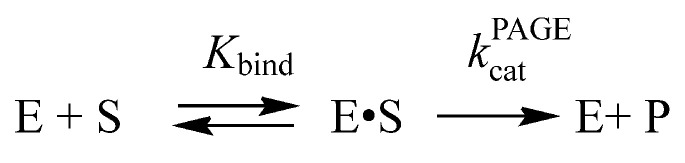
The kinetic mechanism of DNA product accumulation in PAGE analysis. E is AP endonuclease, S denotes the F/N-substrate, P represents the product of the substrate incision reaction, E•S is the catalytically active enzyme–substrate complex, *K*_bind_ means the equilibrium constant of binding step and *k*_cat_^PAGE^ stands for the rate constant of catalytic cleavage of the 5′ phosphodiester bond.

**Figure 5:**
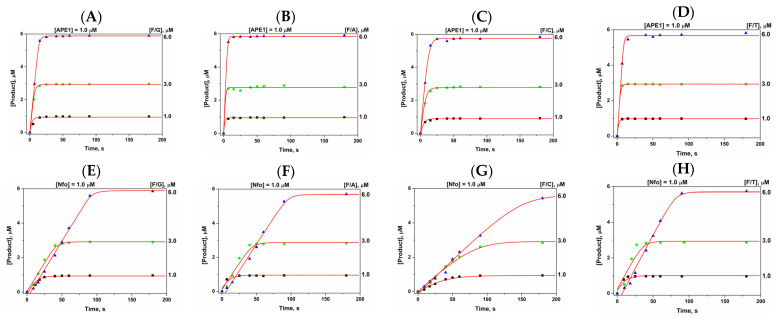
The time course of the accumulation of products during interactions of APE1 (**A**–**D**) or Nfo (E–H) with the F/G- (**A**,**E**), F/A- (**B**,**F**), F/C- (**C**,**G**) or F/T-substrate (**D**,**H**). Concentrations of the AP endonucleases and DNA are shown in the panels.

**Figure 6:**
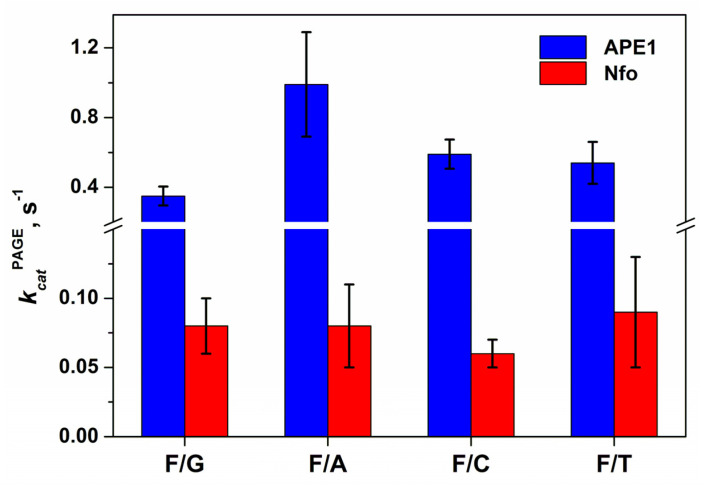
The efficacy of F/N-substrate cleavage by AP endonucleases APE1 and Nfo as evidenced by PAGE analysis.

The rates of the formation of the cleaved DNA product were directly measured for APE1 and Nfo by PAGE with ^32^P-labelled F/N-substrates (Figure 5). Scheme 3 was utilised to describe the resultant kinetic curves. The equilibrium constant was borrowed from the stopped-flow data (Table 1 and Table 2 for APE1 and Nfo, respectively) and was designated as the total binding constant, *K*_bind_. The constant of the irreversible step, *k*_cat_^PAGE^, was estimated by non-linear fitting. The *k*_cat_^PAGE^ values were slightly dependent on the nature of the nucleotide located opposite the F-site and were close to the *k*_cat_ determined in the stopped-flow experiments (Figure 6).

### 2.4. Fluorescent Detection of Opposite-Nucleotide Extrusion in the Course of Catalytic-Complex Formation

**Figure 7:**
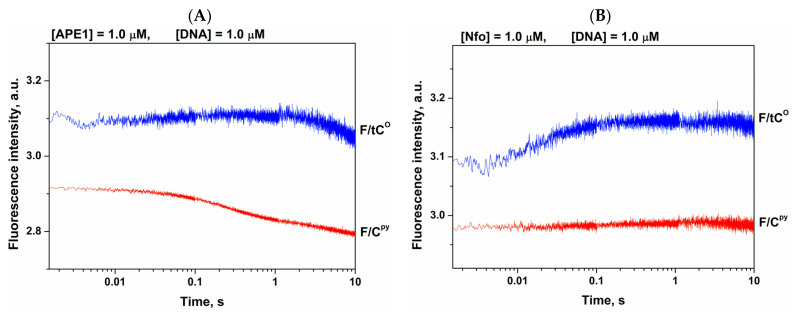
Kinetic curves characterising the opposite-nucleotide extrusion by APE1 (**A**) or Nfo (**B**).

To register the process of opposite-base extrusion from DNA, we used fluorescent analogues of DNA bases, namely, bicyclic cytosine analogue, pyrrolocytosine (3-[β-d-2-ribofuranosyl]-6-methylpyrrolo [2,3-*d*]pyrimidin-2(3H)-one, C^py^) [33,34,35] and a tricyclic cytosine analogue, 1,3-diaza-2-oxophenoxazine (tC^O^) [36,37], which are sensitive to changes of a microenvironment. Typically, the fluorescence of most analogues is quenched by neighbouring nitrogenous bases, and quenching efficiency depends on both the nature of the base and spatial structure of the nucleic acid. Nevertheless, as reported in our previous works, the changes in fluorescence intensity of these bases during protein–DNA interactions are much more complicated than simple quenching by neighbouring bases [38,39]. Therefore, here we performed only a qualitative analysis of fluorescence changes during the interaction of APE1 or Nfo with an F/X-substrate, where X was either C^py^ or tC^O^ (Figure 7). Of note, during initial 0.1 s, fluorescence intensity of tC^O^ declined for Nfo but did not change for APE1. In contrast, C^py^ fluorescence was quite stable in the case of Nfo but significantly dropped for APE1 in the interval from 0.1 to 1.0 s. A comparison of these changes with conformational alterations of the enzymes indicated that the decrease of C^py^ fluorescence intensity by time points 1 s for APE1 or the increase in the tC^O^ signal by time point 0.1 s for Nfo are in good agreement with the second decrease phase of the Trp signal detected for APE1 (Figure 3) and Nfo interacting with the F/C-substrate or F/T-substrate (Figure 4C,D). These findings mean that the opposite-base extrusion proceeds during the second binding step as enzyme-induced conformational transition of the DNA substrate. As evidenced by Trp fluorescence and the conformational alterations of both enzymes, the opposite base influences both substrate-binding steps; therefore, it can be concluded that the nature of the F/N pair determines DNA’s local specific properties, which affect the enzymes in the initial complex.

## 3. Materials and Methods

### 3.1. Protein Expression and Purification

To purify Nfo expressed as a recombinant protein, 1 L of culture (in Luria–Bertani [LB] broth) of *Escherichia coli* strain BL21(DE3) (Invitrogen, France) carrying the pET11a-Nfo construct was grown with 50 μg/mL ampicillin at 37 °C until absorbance at 600 nm (A_600_) reached 0.6–0.7; Nfo expression was induced overnight with 0.2 mM isopropyl-β-d-thiogalactopyranoside. The method for the isolation of wild-type Nfo has been described previously [5]. Human APE1 was purified as described earlier [32,40]. The protein concentration was measured by the Bradford method [41]; the stock solution was stored at −20 °C in 50% glycerol.

### 3.2. Oligodeoxynucleotides (ODNs)

Sequences of the ODNs used in this work are listed in Table 3. The ODNs were synthesised by established phosphoramidite methods on an ASM-700 synthesiser (BIOSSET Ltd., Novosibirsk, Russia) from phosphoramidites purchased from Glen Research (Sterling, VA, USA). Synthetic oligonucleotides were unloaded from solid support according to the manufacturer’s protocols. Deprotected oligonucleotides were purified by high-performance liquid chromatography. Concentrations of oligonucleotides were calculated from their A_260_. ODN duplexes were prepared by annealing modified and complementary strands at a 1:1 molar ratio.

### 3.3. Enzyme Assays by Polyacrylamide Gel Electrophoresis (PAGE)

The enzyme assays involving all the DNA substrates were carried out at 25 °C in reaction buffer [50 mM Tris-HCl pH 7.5, 100 mM KCl, 1.0 mM EDTA, 1.0 mM DDT and 7% of glycerol (*v/v*)]. In the case of APE1, reaction buffer was supplemented with 5.0 mM MgCl_2_. Substrate oligonucleotides were 5′-^32^P-labelled using T4 polynucleotide kinase and [γ-^32^P]ATP. Enzyme concentration was 1.0 μM, and the concentration of a substrate was varied from 1.0 to 6.0 μM. Aliquots of the reaction mixture were withdrawn, immediately quenched with formamide containing 25 mM EDTA and 0.1% xylene cyanol in a volume equal to the aliquot volume, were incubated at 95 °C for 3 min and then were loaded on a 20% polyacrylamide gel containing 7 M urea. Agfa CP-BU X-ray film (Agfa-Geavert, Mortsel, Belgium) was exposed to the gels. The formation of the product was quantified by scanning densitometry in the Gel-Pro Analyzer software, v.4.0 (Media Cybernetics, Rockville, MD, USA). Kinetic parameters were calculated by numerical fitting using DynaFit software (BioKin, Pullman, WA, USA) [42] as described before [43].

### 3.4. Stopped-Flow Fluorescence Measurements

The stopped-flow measurements with fluorescence detection were carried out mostly as described elsewhere [44,45] on an SX.18MV stopped-flow spectrometer (Applied Photophysics Ltd., Leatherhead, UK) equipped with a 150 W Xe arc lamp and an optical cell with 2-mm path length. The dead time of the instrument is 1.4 ms. The fluorescence of Trp was excited at λ_ex_ = 290 nm and monitored at λ_em_ > 320 nm as transmitted by filter WG-320 (Schott, Mainz, Germany). All the experiments were conducted in a buffer consisting of 50 mM Tris-HCl pH 7.5, 100 mM KCl, 1.0 mM EDTA, 1.0 mM DTT and 7% of glycerol (*v/v*). In the case of APE1, reaction buffer was supplemented with 5.0 mM MgCl_2_.

An enzyme was placed in one of the instrument’s syringes and rapidly mixed in the reaction chamber with a DNA substrate from another syringe. The concentrations of the DNA substrate and enzyme are indicated in the figure legends. The reported concentrations of the reactants are those in the reaction chamber after the mixing. Typically, each trace shown in the figures is the average of four or more fluorescence traces recorded in individual experiments. In the figures, for better presentation, the curves were manually moved apart. This procedure does not affect the results of fitting because the background fluorescence is fitted separately for each curve. Kinetic curves were analysed in the DynaFit software (BioKin, Pullman, WA, USA) [42] as described elsewhere [36,37,46,47].

## 4. Conclusions

In spite of extensive studies on AP endonucleases, the molecular basis for their broad substrate specificity and the mechanism of discrimination between modified bases and/or nucleotides as well as undamaged DNA bases are not yet clear. The substrate specificity in relation to a specific site may have something to do with various distortions of local DNA structure around the abasic site, which influence the efficiency of transition to the catalytically active conformation. Another basis for substrate recognition may be related to differences in the efficiency of abasic-site eversion, which is dependent on the nature of the opposite base and/or on local stability of the damaged region in DNA. According to the multiple studies [48,49] on interactions of DNA repair enzymes with their specific site, the common mechanism of damage recognition that ensures high substrate specificity can be subdivided into several steps: the bending of damaged DNA by the enzyme, eversion of the damaged nucleotide from the double helix and verification of the damaged base in the active site of the enzyme through the formation of specific contacts.

Even though it is widely believed that the C-terminal catalytic domain of AP endonucleases accounts for the specific binding to DNA, their N-terminal domains, which substantially vary in size and are not visible in crystal structure, may influence DNA-binding abilities [50,51,52]. Indeed, analysis of APE1 variants with a truncated N-terminal part has revealed that this lysine-rich region may act by stabilising nonspecific association with a nucleic acid through electrostatic interactions [53,54]. A loss of the N-terminal domain influences the stability of both enzyme–substrate and enzyme–product complexes [55,56] but does not affect the rates of initial complex formation or catalysis [31] in the case of the incision of abasic-site–containing DNA. By contrast, this domain affects both the rate of the formation and stability of the initial complex for NIR and 3′→5′ exonuclease activities [10,14,31]. Multiple studies also indicate that the N-terminal domain may functionally interact with various proteins involved in DNA repair [57,58,59,60], transcription [61,62,63] and RNA metabolism [54,64,65,66]. Nevertheless, AP endonuclease Xth from *E. coli* (which belongs to the same structural family as APE1 does), when lacking an N-terminal region, also shows various activities toward different orphan nucleotides [67]. Summarising the literature data, it can be concluded that the N-terminal domain may be required for the preliminary low-affinity binding process during the search for a suitable lesion in DNA or a cleavage site in RNA and during protein–protein interactions. Therefore, in the present study, we compare properties of AP endonucleases by taking into account differences in the main catalytic domain, which is responsible for the specific DNA binding.

In the present work, we analysed the effects of natural nucleotides situated opposite a lesion on the stages of catalytic-complex formation and on the efficacy of DNA cleavage by AP endonucleases: human APE1 and Nfo from *E. coli*, which have completely different protein architectures and structures of the DNA-binding site. A set of model DNA substrates containing the F-site opposite G, A, T or C was used to monitor real-time conformational alterations of the analysed enzymes during their interaction with DNA by means of changes in Trp fluorescence intensity. Our stopped-flow Trp data suggest that catalytic-complex formation in most cases proceeds through a two-step kinetic mechanism of binding.

These findings permit the conclusion that kinetic parameters of the binding steps resulting in a catalytically competent enzyme–substrate complex are significantly different among the tested F/N-substrates, indicating the importance of the opposite-base nature. Initial-DNA-binding constants and total binding constants typically decrease in the order F/G > F/A ≥ F/T > F/C.

The absence of direct contacts of the enzyme with the base located opposite to the damage suggests that the conformational DNA rearrangements induced by the enzymes are dependent on the internal interactions of the nucleotide located opposite the lesion with the neighbouring bases in the double DNA helix. It was demonstrated that catalytic rate constants are mostly independent from the nature of the opposite base. Therefore, in the course of enzyme conformational transitions, the efficacy of damaged-DNA cleavage with respect to various nucleotides in the complementary strand is controlled mostly during initial-complex formation. On the contrary, in the Nfo interaction with an F/N-substrate, the extrusion of purine bases at the second step of the interaction proceeds much faster than that of pyrimidine bases thereby eliminating this step from the kinetic scheme.

Taken together, our results for the first time show that structurally different AP endonucleases Nfo and APE1 utilise a common strategy of damage recognition, which involves the formation of an initial enzyme–substrate complex and its subsequent transformation to the catalytic state. It could be concluded that local structural properties of DNA near damaged nucleotide is crucial for efficient initial lesion-sensing and subsequent formation of the catalytic complex independently on the structure of the enzyme active site.

## Figures and Tables

**Table 1:** The rate and equilibrium constants of the interaction of APE1 with an F/N-substrate ^a^.

Constants	F/G ^b^	F/A	F/C	F/T
*k*_1_, M^−1^s^−1^	(90 ± 10) × 10^6^	(37 ± 4) × 10^6^	(5.5 ± 1.2) × 10^6^	(58 ± 7) × 10^6^
*k_−_*_1_, s^−1^	10.1 ± 4.3	66 ± 9	110 ± 10	120 ± 10
*K*_1_, M^−1^	(8.9 ± 4.8) × 10^6^	(0.6 ± 0.1) × 10^6^	(0.05 ± 0.01) × 10^6^	(0.5 ± 0.1) × 10^6^
*k*_2_, s^−1^	43.1 ± 8.5	3.9 ± 0.3	11.5 ± 2.7	10.0 ± 2.0
*k_−_*_2_, s^−1^	7.1 ± 1.6	0.47 ± 0.02	0.52 ± 0.02	0.88 ± 0.06
*K*_2_	6.1 ± 2.6	8.3 ± 1.0	22 ± 6	11 ± 3
*K*_bind_, M^−1^	(63 ± 57) × 10^6^	(5.6 ± 1.5) × 10^6^	(1.2 ± 0.5) × 10^6^	(6.0 ± 2.7) × 10^6^
*k*_cat_, s^−1^	0.3 ± 0.1	0.16 ± 0.01	0.10 ± 0.01	0.23 ± 0.03
*K*_P_, M	(7.2 ± 0.4) × 10^−6^	(1.0 ± 0.2) × 10^−6^	(11 ± 2) × 10^−6^	(32 ± 5) × 10^−6^

^a^ *K*_i_ = *k*_i_/*k*_−i_, *K*_bind_ = *K*_1_ + *K*_1_ × *K*_2._ ^b^ from ref. [29].

**Table 2:** The rate and equilibrium constants of the interaction of Nfo with an F/N-substrate ^a^.

Constants	F/G	F/A	F/C	F/T
*k*_1_, M^−1^s^−1^	(151 ± 2) × 10^6^	(360 ± 10) × 10^6^	(150 ± 10) × 10^6^	(360 ± 10) × 10^6^
*k*_−1_, s^−1^	1.9 ± 0.2	30 ± 1	270 ± 10	235 ± 10
*K*_1_, M^−1^	(80 ± 9) × 10^6^	(12 ± 1) × 10^6^	(0.55 ± 0.06) × 10^6^	(1.5 ± 0.1) × 10^6^
*k*_2_, s^−1^	-	-	9.1 ± 0.2	101 ± 2
*k*_−2_, s^−1^	-	-	4.4 ± 0.1	28 ± 1
*K*_2_	-	-	2.1 ± 0.1	3.6 ± 0.2
*K*_bind_, M^−1^	(80 ± 9) × 10^6^	(12 ± 1) × 10^6^	(1.7 ± 0.2) × 10^6^	(6.9 ± 0.8) × 10^6^
*k*_cat_, s^−1^	0.10 ± 0.01	0.18 ± 0.02	0.27 ± 0.01	0.23 ± 0.01
*K*_P_, M	(11 ± 1) × 10^−6^	(5.8 ± 0.4) × 10^−6^	(6.9 ± 0.4) × 10^−6^	(2.7 ± 0.1) × 10^−6^

^a^ *K*_i_ = *k*_i_/*k*_−i_, *K*_bind_ = *K*_1_ + *K*_1_ × *K*_2_.

**Table 3:** DNA sequences and structure of the lesion introduced into the DNA duplexes.

Shorthand	Sequences of DNA Duplexes
F/N-substrate N = G, A, T, C	5’-CTCTCFCCTTCC-3’ 3’-GAGAGNGGAAGG-5’

F is (2*R*,3*S*)-2-(hydroxymethyl)-3-hydroxytetrahydrofuran (an AP-site analogue).

## Data Availability

Data is contained within the article.
